# Using a Non-Image-Based Medium-Throughput Assay for Screening Compounds Targeting *N*-myristoylation in Intracellular *Leishmania* Amastigotes

**DOI:** 10.1371/journal.pntd.0003363

**Published:** 2014-12-18

**Authors:** Daniel Paape, Andrew S. Bell, William P. Heal, Jennie A. Hutton, Robin J. Leatherbarrow, Edward W. Tate, Deborah F. Smith

**Affiliations:** 1 Centre for Immunology and Infection, Department of Biology, University of York, York, United Kingdom; 2 Institute of Chemical Biology, Department of Chemistry, Imperial College London, London, United Kingdom; The Ohio State University, United States of America

## Abstract

We have refined a medium-throughput assay to screen hit compounds for activity against *N*-myristoylation in intracellular amastigotes of *Leishmania donovani*. Using clinically-relevant stages of wild type parasites and an Alamar blue-based detection method, parasite survival following drug treatment of infected macrophages is monitored after macrophage lysis and transformation of freed amastigotes into replicative extracellular promastigotes. The latter transformation step is essential to amplify the signal for determination of parasite burden, a factor dependent on equivalent proliferation rate between samples. Validation of the assay has been achieved using the anti-leishmanial gold standard drugs, amphotericin B and miltefosine, with EC_50_ values correlating well with published values. This assay has been used, in parallel with enzyme activity data and direct assay on isolated extracellular amastigotes, to test lead-like and hit-like inhibitors of *Leishmania N*-myristoyl transferase (NMT). These were derived both from validated in vivo inhibitors of *Trypanosoma brucei* NMT and a recent high-throughput screen against *L. donovani* NMT. Despite being a potent inhibitor of *L. donovani* NMT, the activity of the lead *T. brucei* NMT inhibitor (DDD85646) against *L. donovani* amastigotes is relatively poor. Encouragingly, analogues of DDD85646 show improved translation of enzyme to cellular activity. In testing the high-throughput *L. donovani* hits, we observed macrophage cytotoxicity with compounds from two of the four NMT-selective series identified, while all four series displayed low enzyme to cellular translation, also seen here with the *T. brucei* NMT inhibitors. Improvements in potency and physicochemical properties will be required to deliver attractive lead-like *Leishmania* NMT inhibitors.

## Author Summary

We have developed an assay for screening test compounds for their ability to kill intracellular amastigotes of *Leishmania* parasites, causative agents of human leishmaniasis. The assay is based on freeing amastigotes from infected macrophages by mild detergent lysis and measuring the number of parasites following their extracellular replication by a fluorescence-based method. The validity of the assay has been confirmed using the gold standard drugs, Amphotericin B and Miltefosine, which kill parasites at highly reproducible concentrations. Our results show that this assay is easily transferable between laboratories, can be adapted to specific applications and used to test any parasite species or strain, and does not rely on genetically-modified parasites. These features will enable its use in screening isolates taken directly from patients, vectors or reservoir hosts. We used this assay, in parallel with enzyme activity data, to test lead-like and hit-like inhibitors of a validated target enzyme, *Leishmania N*-myristoyltransferase (NMT). Compounds from two of four newly-identified *Leishmania* NMT-selective hit series displayed host cell cytotoxicity, while all four series displayed low translation of enzyme to cellular activity in analysis of intracellular parasite viability. Improvements in potency and physicochemical properties will be required to deliver attractive lead-like *Leishmania* NMT inhibitors.

## Introduction

The Leishmaniases, together with Human African Trypanosomiasis (HAT) and Chagas disease, are caused by kinetoplastid parasites of the TriTryp group (*Leishmania* spp., *Trypanosoma brucei* and *T. cruzi* respectively [1]–[3] and described as neglected tropical diseases [4]. All of these infections are diseases of poverty and cause severe impact, as measured in disability adjusted life years (DALY), in endemic countries (91 in total for the Leishmaniases, including countries in east and northern Africa, the Middle East, the Indian sub-continent and Central and South America [3]). They have also received limited funding for research and development of new drugs [4] although there are currently “repurposed” drugs in clinical or preclinical trials for all three disease groups e.g. fexinidazole for visceral leishmaniasis (VL) and HAT (acute and CNS stages) [5], [6]; the antifungal lanosterol-14 alpha-demethylase inhibitors, posaconazole [7] and E1224 (a prodrug of ravuconazole [8], [9]), for Chagas disease (see [4] and www.dndi.org/ for further details). Despite this recent encouraging progress, there is an urgent need to develop more potential therapeutics and especially, to identify new chemical entities which are orally available and fast acting for the treatment of these diseases. The aim is to cure with a single oral daily dose over a 10 day course in the case of VL and HAT, a challenging target-product profile particularly for the intracellular (amastigote) *Leishmania* parasite in the host.

Small molecule screens with *Leishmania spp.* have often been performed with the easily cultured but less clinically-relevant extracellular insect (promastigote) form of the parasite [10]–[13]. Axenic amastigotes, adapted to replicate at acidic pH and elevated temperature as extracellular parasites, are also used as a screening resource [14], [15] but are not ideal as they are not derived from the parasitophorous vacuole (PV), the intracellular compartment in which amastigotes reside within host cells [16]. Indeed, the differing gene expression and proteomic profiles presented by promastigotes, axenic amastigotes and intracellular amastigotes of several *Leishmania* species suggest differing molecular profiles during parasite stage differentiation. For example, the mRNA expression profiles of *L. mexicana* promastigotes and axenic amastigotes are remarkably similar whereas when either is compared to the mRNA profile of lesion-derived amastigotes, different mRNAs are significantly up- or down-regulated [17]. Similarly, analysis of global mRNA expression profiles of *L. infantum* axenic and intracellular amastigotes isolated from cultured human (THP-1) macrophages reveal very few differentially expressed genes in common between the stages [18]. At the protein level, comparison of promastigotes with intracellular amastigotes in *L. mexicana* reveals several proteins upregulated solely in amastigotes, including enzymes linked to respiration/energy metabolism, fatty acid metabolism and protein synthesis, and proteins involved in stress responses [19]. Upregulated fatty acid metabolism has also been described in comparisons of lesion-derived *L. mexicana* amastigotes and dividing promastigotes [20].

These observations confirm that small compound testing should ideally be focused on the clinically-relevant parasite stage, the intracellular amastigote. This conclusion is supported by a recent study comparing compound efficacy against extracellular promastigotes and intracellular amastigotes; only a small number of those compounds active against the extracellular life cycle stages were also active against the intracellular stage [21]. In addition, progressing a promastigote screening cascade towards intracellular screening has been shown to not only identify false positives but also exclude compounds that are active against the relevant intracellular stage [22]. It is possible that the study design may have selected for compounds active against differentiating parasites rather than true intracellular amastigotes in this case; compounds were added immediately after removing free parasites from macrophages infected over a period of 4 h with *L. donovani* promastigotes, a stage at which amastigote differentiation would not have been complete. As an additional complication, compounds that are active against one *Leishmania* species may not be active against another, if the observed differences in gene expression profiles [23] are reflected in their proteomes and metabolomes [24], [25].

High-throughput image-based phenotypic screens of intracellular amastigotes have gained support as the current preferred approach for drug discovery against *Leishmania spp.* [21], [22] and undoubtedly provide a superior approach for screening small compounds and conducting lead-optimization. However, sophisticated imaging resources are not readily available in many lab settings, preventing widespread adoption of these techniques. In addition, some image-based assays rely on genetically-modified parasites carrying a selectable marker, a feature that can compromise the identification of new compounds by conferring resistance itself, as reported for neomycin and paromomycin in *Saccharomyces cerevisiae* [26]. The generation of mutants without selectable markers should overcome this problem [27]. The expression levels of fluorescent or bioluminescent reporters encoded on episomes is dependent on their episomal copy number but this can also vary in a clonal population [28], potentially affecting the read-out to determine the compound efficacy. Similarly, genetic modification of parasites might change their biological properties and subsequently, their sensitivity to test compounds [29].

Jain and colleagues [30] recently approached the lack of a non–image based method for intracellular amastigote screening by developing an assay in which *L. donovani* promastigotes were used to infect cultured THP-1 macrophages, differentiated amastigotes subsequently released by SDS lysis and, following transformation back to promastigotes and growth for two days, quantified using Alamar blue. In parallel work, driven by the need to advance a specific drug development project for the leishmaniases, we have adopted a similar assay, although using clinically-relevant amastigotes for infection of bone marrow-derived macrophages together with saponin lysis, and subsequently validated the method using the gold standard drugs against VL, amphotericin B and miltefosine.

Utilising this assay, we have extended work on the development of myristoyl-CoA:protein *N*-myristoyltransferase (NMT) as a promising candidate for the development of anti-kinetoplastid drugs [31]–[34]. In collaboration with Pfizer, we recently published the results of a high-throughput screen against *L. donovani* NMT that resulted in the identification of four chemically diverse and selective series [35]. Here, we present results generated with the novel NMT inhibitors identified in the Pfizer screen [35], together with several *T. brucei* NMT inhibitors [36], [37]. Several of the latter compounds were reported to have similar activity against *L. major* NMT and we have also found them to be potent inhibitors of *L. donovani* NMT. Structural biology studies on the compounds derived from the Pfizer screen have been published recently [38]; structure-activity-relationships and synthetic details are being published separately [39].

## Materials and Methods

### Animals and parasites

BALB/c mice were obtained from Charles River (Margate, UK). Rag-2^−/−^ mice were bred in-house, housed under specific pathogen-free conditions and used at 6–12 weeks of age. The Ethiopian strain of *Leishmania donovani* (MHOM/ET/67/HU3, also known as LV9) was maintained by serial passage in Rag-2^−/−^ mice. Amastigotes were isolated from infected spleens by homogenization and saponin lysis, as previously described [40]. Mice were infected with 3×10^7^ *L. donovani* amastigotes intravenously (i.v.) via the tail vein in 200 µl of RPMI 1640 (GIBCO, Paisley, UK). All experiments were approved by the University of York Animal Procedures and Ethics Committee and performed under UK Home Office licence (‘Immunology and Immunopathology of Leishmaniasis’ Ref # PPL 60/4377).

### *In vitro* infection

Macrophages (BMDM) were differentiated from bone marrow of 6–8 weeks old female BALB/c mice as described previously [41] and plated out at 4.2×10^4^ cells/per well in 96-well plates. BMDMs were adhered for at least 4 h and infected overnight at a multiplicity of infection of 15 with freshly isolated *L. donovani* amastigotes (see above). BMDMs were maintained in DMEM supplemented with 4 mM L-Glutamine (both GIBCO, Paisley, UK) and 4% L929-cell conditioned medium. All experiments were performed at 37°C and 5% CO_2_.

### Reference compounds and determination of parasite burden

The anti-leishmanial reference drugs amphotericin B (Fungizone, kindly provided by Vanessa Yardley, London School of Hygiene and Tropical Medicine) and miltefosine (Sigma, #M9198) were used, the former reconstituted according to the manufacturer's instructions. Miltefosine was dissolved in deionised water. Compounds were prepared at 2× concentration in a 7-point threefold dilution series and tested in triplicate. The highest concentrations used were 30 µM (miltefosine) and 1.2 µM (amphotericin B). Infected macrophages were incubated with the compounds for 72 h. Infected and uninfected cells were then washed twice with DMEM before lysis with 100 µl 2 mg·ml^−1^ saponin in DMEM for 5 min at room temperature (RT). Lysis was stopped by addition of 100 µl undiluted fetal calf serum (FCS).

Plates were centrifuged at 2200×g (Heraeus Multifuge 3SR Plus) for 5 min at RT and the medium carefully removed. For all manipulations of this type, as much as possible of the supernatant was carefully but swiftly removed with an 8-channel pipette, placing the pipette tips into the corners of the wells in an angled plate and quickly aspirating until virtually all liquid was removed. Fresh medium was quickly replaced to prevent cells drying out. In this case, 200 µl RPMI 1640 medium (#22409-015, GIBCO, Paisley, UK) supplemented with 20% heat-inactivated FCS, 100 µM adenine, 20 mM 2-[N-morpholino] ethanesulphonic acid (pH 5.5), 5 µM hemin, 3 µM biopterin, 1 µM biotin, penicillin (100 U/ml) and streptomycin (100 µg/ml) were then added, at final pH 6.7, and incubation continued at 26°C. After 96 h, 20 µl Alamar blue (Trek Diagnostics) was added and incubation continued at 26°C for a further 6 h or until the Alamar blue was reduced sufficiently (as determined visually by its conversion from oxidised state blue to reduced state pink). Further reduction was stopped by addition of 20 µl 8% formaldehyde solution. Fluorescence was measured using a POLARstar Optima (BMG Labtech) plate reader (ex. 544 nm, em. 590 nm) with the lid removed.

Drug dilutions for the extracellular assays were prepared in the same way but in supplemented RPMI medium. Assays were performed in triplicate with 4×10^5^ *L. donovani* amastigotes (freshly isolated as described above) per well per 96-well plate, plating 100 µl of parasite suspension per well first and then adding the drug dilution to be tested. Sealed 96 well plates were then placed at 26°C. After 72 h, 20 µl Alamar blue was added and the fluorescence measured 6 h later.

Host cell cytotoxicity was determined by seeding out as above; compounds were added at 2× concentration in a 7-point threefold dilution series and tested in duplicate. After 72 h, 1/10 vol Alamar blue was added and the fluorescence measured 6 h later. To determine LD_50_ values, the following highest concentrations were used based on the *L. donovani* NMT potency or, if necessary, lowered due to compound insolubility at a higher concentration: 150 µM – IMP-0000556; 90 µM - IMP-0000197, DDD100887 and DDD86211; 45 µM - DDD85646, IMP-0000083, IMP-0000195.

Drug activities were determined by calculating the percentage of the measured fluorescence values of treated compared to untreated cells. GraphPad Prism (ver. 5) was used to plot dose response curves, to calculate EC_50_ values by non-linear regression with variable slope, to calculate correlation coefficients of curves and for statistical analysis. Table 1 lists the mean EC_50_ values and standard deviation for each experiment, calculated using GraphPad Prism (ver. 5).

**Table 1:** Activity of gold standard drugs after 3-day treatment against intra- and extracellular *L. donovani* amastigotes.

	EC_50_ ± SD intracellular *L. donovani* amastigotes	EC_50_ ± SD extracellular *L. donovani* amastigotes*
**Amphotericin B**	30±9 nM	50±11 nM
**Miltefosine**	1.38±0.06 µM	7.85±0.5 µM

*Assay was set up with amastigotes isolated from spleens of infected Rag2−/− mice with subsequent incubation at 26°C. All assays were run in triplicate with biological repeats (intracellular amphotericin B, n = 4; intracellular miltefosine, n = 3; extracellular amphotericin B, n = 3; extracellular miltefosine, n = 2).

## Results

### Establishing the *L. donovani* amastigote assay

**Figure 1:**
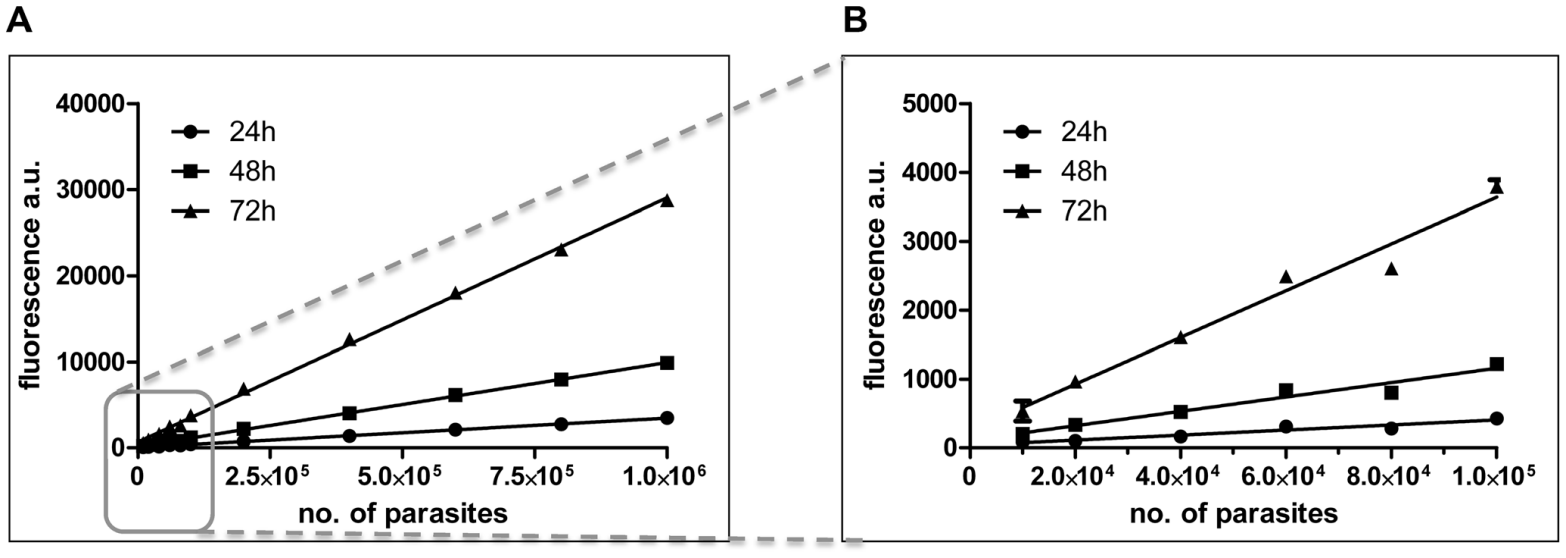
Linear correlation of the number of *L. donovani* amastigotes and the reduction of Alamar blue. *L. donovani* amastigotes were isolated from the spleen of an infected Rag-2 −/− mouse. Up to 10^6^ (A) or 10^5^ (B) parasites were suspended in 200 µl/well in supplemented RPMI, Alamar blue was added and the plate incubated at 26°C. At the indicated time points, the fluorescence per well was determined and plotted over the initial number of parasites/well. The correlations between Alamar blue conversion and the initial parasite numbers are highly significant: P<0.0001 as shown by linear regression analysis (R^2^24h– 0.9979, R^2^48h – 0.9991, R^2^72h – 0.9984).

To avoid reliance on fluorescent/luminescent parasites, sophisticated microscopy equipment or counting parasites on Giemsa-stained slides, we developed an assay for drug-testing similar to that already described [30] in which surviving parasites are released from infected macrophages, allowed to differentiate into promastigotes and then enumerated. In our assay, bone marrow-derived macrophages are infected with *L. donovani* amastigotes and incubated for 72 h in the presence or absence of test or control compounds. Macrophages are then subject to conditional lysis with saponin, a milder treatment than the 0.05% SDS used by Jain et al. [30], followed by incubation for up to 4 days in promastigote medium at 26°C. During this time, the amastigotes trapped in the macrophage debris transform to promastigotes and these break free (due to flagellar motion) and commence replication. In the presence of Alamar blue, its active component, resazurin, is then reduced to resorufin by the metabolically active parasites. This reduction is directly dependent upon parasite numbers (see Fig. 1). The transformation from amastigote to promastigote and subsequent proliferation is necessary to amplify the signal obtained by the Alamar blue reduction. This allows determination of the parasite burden - that is, the respective parasite load on the day of lysis, assuming an equal proliferation rate irrespective of initial parasite numbers.

Firstly, a direct correlation was established between parasite numbers after transformation and the resulting fluorescence from the reduced Alamar blue. This was done by seeding freshly isolated splenic amastigotes in supplemented RPMI into a 96-well plate. Alamar blue was added and the parasites incubated at 26°C. Transformation occurred over time and the resulting promastigotes started to replicate, resulting in Alamar blue reduction. Fluorescence was measured every 24 h to monitor this reduction of the dye relative to the parasite numbers per well. As shown in Fig. 1, the reduction of Alamar blue was directly proportional to the numbers of viable cells over at least two orders of magnitude (initial cell numbers 10^4^–10^6^) for at least 72 h (Fig. 1). This finding confirms the assumption that the proliferation rate is equivalent between samples, irrespective of the initial number of parasites.

Linearity was tested with up to 10^6^ *L. donovani* amastigotes (Fig. 1), which in our hands reflected an idealized condition as these cell numbers were not achieved upon conditional lysis of the macrophages. This correlation should however be verified prior to implementation of this assay as it is dependent on the parasite strain/s as well as the macrophage cell lines used. It is important to stress that the protocol described here is only evaluating the anti-leishmanial activity of compounds against the intracellular lifecycle stage. Upon lysis and freeing of amastigotes, the transformation to promastigotes is not affected by the compounds as these are no longer present at this stage of the protocol.

**Figure 2:**
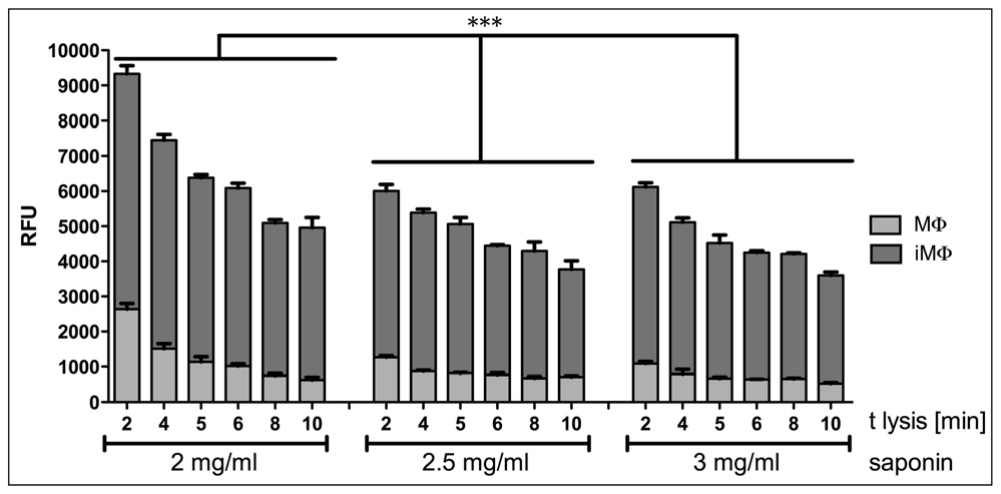
Release of parasites from infected macrophages in 96-well plates. Bone marrow derived macrophages were infected overnight with *L. donovani* amastigotes. Cells were incubated for a further 3 days and then treated with DMEM containing the indicated concentration of saponin. Supplemented RPMI was added to the released amastigotes and plates were further incubated for 4 days at 26°C before addition of Alamar blue and subsequent measurement of fluorescence in a plate reader. The survival of freed amastigotes is dependent on the concentration of saponin used and the time of lysis. A 5-fold greater signal of reduced Alamar blue from freed amastigotes over uninfected macrophages was achieved with 2 mg·ml-1 saponin for 5 min; this concentration was used for all other experiments performed. All samples were run in triplicate; one representative experiment is shown. MΦ, uninfected macrophages; iMΦ, infected macrophages. *** denotes P<0.0001 as determined by a two-way ANOVA.

Secondly, uninfected and infected macrophages were lysed with varying amounts of saponin (2, 2.5 and 3 mg·ml^−1^), a milder detergent compared to SDS, dissolved in non-supplemented DMEM. As shown in Fig. 2, in our hands amastigotes were most effectively freed from macrophages, with limited survival of the latter, using 100 µl of 2 mg·ml^−1^ saponin and incubation for 5 min, prior to lysis termination by addition of 100 µl of undiluted FCS. Analysis using two-way ANOVA with Bonferoni post test on the fluorescence intensity difference between infected and uninfected macrophages showed that significantly more amastigotes were freed from macrophages when using 2 mg·ml^−1^ saponin compared to higher concentrations; there was no significant difference between 2.5 and 3 mg·ml^−1^ saponin lysis. Using this lysis method, a 5-fold greater signal from freed amastigotes over live macrophages was achieved. To determine the lysis efficiency (i.e. the background level) and to compensate for inter-experiment variation, uninfected macrophages were monitored in all experiments carried out using this method.

### Validating the amastigote assay with known drugs

**Figure 3:**
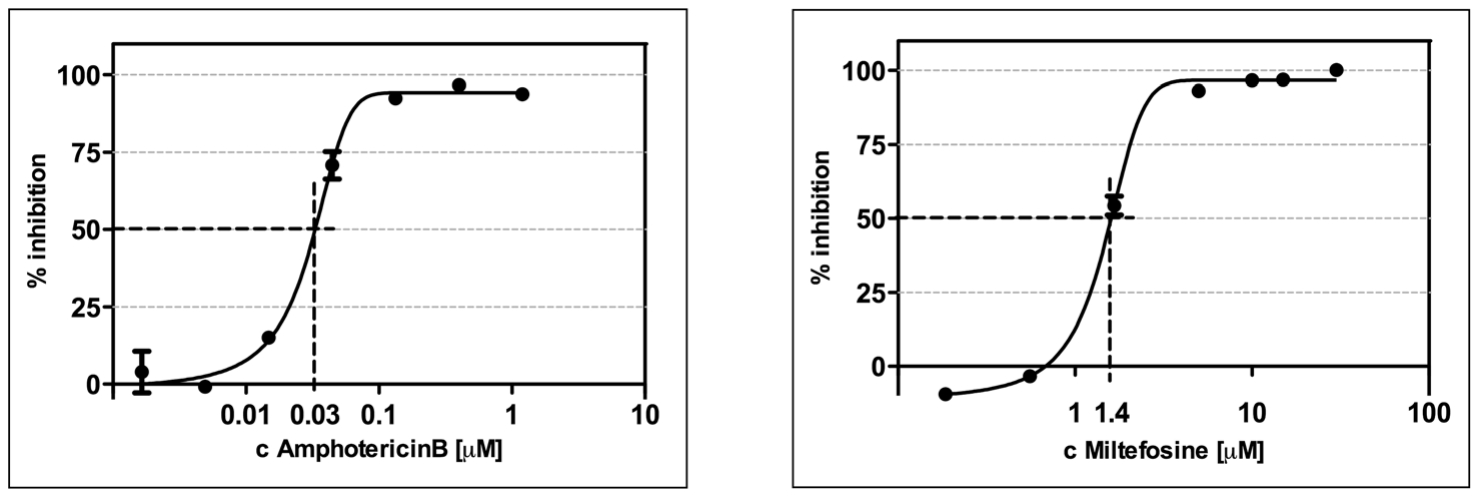
Dose response curves for amphotericin B and miltefosine against intracellular *L. donovani* amastigotes. Parasites were released by conditional lysis with saponin from bone marrow derived macrophages of BALB/c mice after 72 h incubation with anti-leishmanial standard drugs. Using this method, the measured activities of amphotericin B (EC_50_ 29 nM) and miltefosine EC_50_ (1.45 µM) were reproducible and comparable to reported values against intracellular L. donovani (43–45). Representative plots for amphotericin B (n = 4) and miltefosine (n = 3) are shown. (R^2^_AmphoB_ – 0.9905, R^2^_Mil_ – 0.9963).

To confirm the efficacy of this assay, that uses primary macrophages infected with freshly isolated amastigotes rather than a macrophage-like cell line infected with promastigotes [30], dose response curves and EC_50_ values were generated for amphotericin B and miltefosine (the gold standard drugs for the treatment of VL [4]). The EC_50_ values were 30 nM and 1.38 µM respectively, correlating well with the data described by other groups [42]–[44]; see Fig. 3 and Table 1.

Amphotericin B and miltefosine were also tested on amastigotes freshly isolated from mouse spleens, thereby avoiding any in vitro macrophage infection. These assays, referred to as the “extracellular assays” hereafter, resulted in EC_50_ values of 50 nM and 7.85 µM respectively (dose response curves not shown, Table 1) and were also in good alignment with values described elsewhere [43], [45].

### Testing small compounds inhibitory to NMT

**Figure 4:**
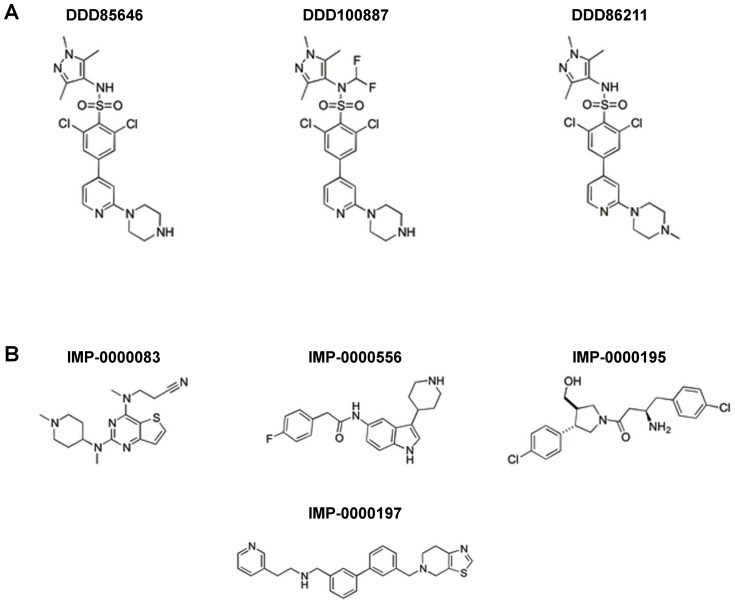
Chemical structures of NMT inhibitors. (A) Resynthesised *T. brucei* inhibitors identified by the Dundee Drug Discovery Unit as also inhibiting *L. major* NMT [31], [37]. Results summarized in Table 2. (B) Selective inhibitors identified by high throughput screening against *L. donovani* NMT [35]. Results summarised in Table 3.

**Figure 5:**
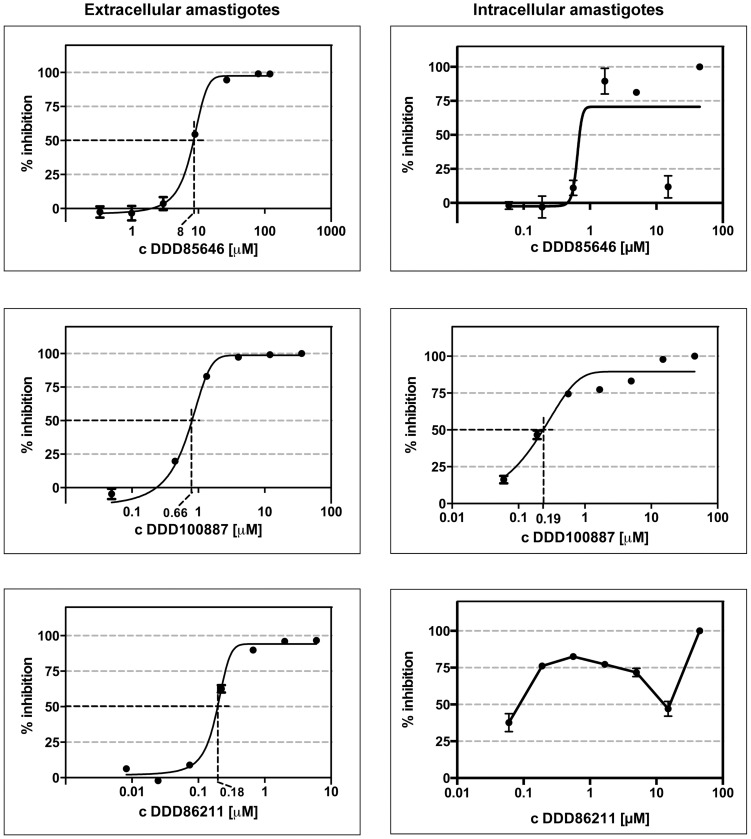
Cellular activity of compounds with good potency in the extracellular but varying potency in the intracellular model. All three compounds tested showed cellular activity on the intracellular and extracellular *L. donovani* amastigotes. When tested against intracellular amastigotes, only DDD100887 generated a reproducible dose-response curve. It was not possible to generate dose-response curves for DDD85646 and DDD86211 as both compounds resulted in an reproducible “activity valley” at higher concentrations before resulting in complete killing of the parasites at the highest concentration tested (see text for details). One representative experiment is shown for each compound. The correlation coefficients for the extracellular amastigotes were R^2^>0.98, with almost all very close to or >0.99. The correlation coefficient for intracellular DDD100887 is R^2^ 0.9212.

NMT inhibitory compounds were tested against *L. donovani* amastigotes in both the extracellular and intracellular assays described above. The focus was on the small compounds identified by high throughput screening against *L. donovani* NMT and found to have selectivity over the human NMTs (Fig. 4B) [35]. In addition, we profiled several examples from a series of *T. brucei* NMT inhibitors identified by the Dundee Drug Discovery Unit, since this series has been reported to also inhibit *L. major* NMT [36]. The original small molecule showing the highest activity against the NMT of bloodstream stages of *T. brucei* in vivo (DDD85646; Fig. 4A), identified by Frearson et al. [31], and two further compounds derived from this TbNMT inhibitor [37] (Fig. 4A, Fig. 5 and Table 2) were resynthesized and tested. The activities of all of these resynthesised compounds were confirmed by testing on purified *L. donovani* NMT (IC_50_ values in Tables 2 and 3), using the assay described by Goncalves and colleagues [46]. The figures generated were in agreement with those reported previously [35], [36]. The compounds were then progressed to evaluation of their cellular activity.

**Table 2:** Activity values/properties of NMT inhibitors from DDD85646 series.*

	IC_50_ *L. donovani* NMT	EC_50_ extracellular *L. donovani* amastigotes	EC_50_ intracellular *L. donovani* amastigotes	LD_50_ bone marrow derived macrophages	pKa	cLogP	Hydrogen bond donors
**DDD85646** 1	3.6 nM	8 µM	n.p.	>45 µM	8.8	2.0	2
**DDD100887** 2	20 nM	660 nM	190 nM	23 µM	8.8	4.2	1
**DDD86211** 2	7.3 nM	180 nM	n.p.	>45 µM	6.5	2.4	1

1 resynthesised DDD85646 (Ref 31).

2 resynthesised from WO2010/026365 [Ref 37].

*structures shown in Fig. 4A.

n.p. – not possible.

**Table 3:** Activity values/properties of NMT inhibitors identified in Pfizer high-throughput screen.^1^^,^ 2

	IC_50_ *L.don.* NMT	EC_50_ extracellular *L. donovani* amastigotes	LD_50_ bone marrow derived macrophages	pKa	cLogP	Hydrogen bond donors
**IMP-0000083 (analogue of PF-00349412)**	281 nM	24 µM	>45 µM	8.4	2.6	0
**IMP-0000556 (PF-03393842)**	316 nM	no activity up to 30 µM	>45 µM	10.0	3.1	2
**IMP-0000195 (PF-03402623)**	77 nM	12 µM	12 µM	8.9	2.9	2
**IMP-0000197 (PF-00075634)**	914 nM	16 µM	50 µM	9.8	4.5	1

1 Ref 35.

2 structures shown in Fig. 4B.

Hit compounds derived from the high-throughput screen were resynthesized and their enzyme activity confirmed in our assays. A range of different profiles were observed in the cellular assays with IMP-0000556 showing no cellular activity up to 30 µM whereas IMP-0000197 gave an EC_50_ of 16 µM in the extracellular assay. IMP-0000195, the most potent LdNMT inhibitor from the HTS screen, had a moderate cellular activity (EC_50_ 12 µM) but was equally toxic to macrophages. Of the HTS-derived compounds, IMP-0000083 (a close analogue of the Pfizer hit) has the most promising profile. Although ∼4-fold less potent in its inhibition of LdNMT than IMP-0000195, the activity difference was reduced at the cellular level, while showing no toxicity to host cells (Table 3).

The re-synthesised version of the original *T. brucei* NMT inhibitor, DDD85646, as well as derivatives thereof (DDD86211 and DDD100887), were tested in both the extracellular assay and the intracellular assay (Fig. 5). DDD85646 was the most potent LdNMT inhibitor in the enzyme assay, followed by DDD86211 and DDD100887. However, at the cellular level, DDD85646 was the least active (EC_50_ 8 µM in the extracellular amastigote assay). The cellular activity of DDD86211 against extracellular amastigotes (EC_50_ 180 nM) was impressive, while DDD100887 was also effective (EC_50_ 660 nM). All three compounds also had some activity against intracellular *L. donovani* amastigotes (Fig. 5 and Table 2). However, only compound DDD100887 generated a genuine dose-response curve with an EC_50_ value of 190 nM. In comparison, DDD85646 and DDD86211 caused an initial concentration-dependent increase in anti-leishmanial activity followed by a reproducible decrease at 15 µM. At the highest concentration tested, 45 µM, both compounds exerted a strong cytotoxic effect and all amastigotes were cleared from the macrophages (Fig. 5). For both DDD85646 and DDD86211, this “activity valley” was a reproducible effect over successive experiments.

## Discussion

We have developed a robust assay to screen for inhibitory compounds active against intracellular *Leishmania*, independent of automated fluorescent microscopy or the time-consuming counting of parasites on Giemsa-stained slides. It is similar to a previously reported assay [30] but uses isolated splenic amastigotes rather than cultured promastigotes for the initial infection of primary macrophages and subsequent reproducible lysis by the mild detergent saponin over a 5 min period, rather than harsher SDS treatment for 30 sec. We have focused on *L. donovani* as our test organism here, given its importance as the causative agent of the most severe form of leishmaniases, but other *Leishmania* species and/or host cells could easily be used in this assay format.

Key steps in optimising the assay included determining the best lysis conditions for parasite release while retaining only marginal background signal from any surviving macrophages. For the *L. donovani*/bone marrow-derived macrophage model used here, lysis with 2 mg·ml^−1^ saponin in DMEM for 5 min at room temperature gave the best results, with amastigote integrity compromised at higher saponin concentrations (Fig. 2). After macrophage lysis and centrifugation, the amastigote-containing supernatant was supplemented with RPMI medium and incubated at 26°C, triggering amastigote to promastigote transformation and subsequent parasite proliferation. This second stage of the assay was critical for amplification of the signal over background; the number of amastigotes present on the day of lysis was insufficient to achieve this result. By performing this incubation step, the resulting signal is dependent on the initial amastigote number as long as the differentiated promastigotes are in the logarithmic growth phase (Fig. 1). In our hands, this phase continued for at least 4 days, by which time the signal was sufficiently amplified to generate highly reproducible results. While an assay requiring 9 days for completion may be considered non-optimal, this protocol allows a number of compounds to be tested sequentially (possible at medium through-put in culture wells) and has the advantage that counting of parasites is not required rather than the admittedly more exacting counting of Giemsa-stained amastigotes in infected macrophages on slides. Thus results between laboratories should be subject to less variability. A further advantage of this method is its lack of dependence on genetically modified parasites, facilitating compound testing on both resistant and sensitive parasite field isolates.

We want to highlight that both this assay and the Jain assay [30] are apparently good alternatives for screening inhibitory compounds active against intracellular *Leishmania* parasites, being independent of automated fluorescent microscopy or Giemsa-staining. Our assay is straightforward to establish, highly reproducible and has the potential for semi-automation to facilitate higher through-put screening.

Validation of this assay has been achieved using the anti-leishmanial gold standard drugs, amphotericin B and miltefosine. The resulting EC_50_ values are in good agreement with those reported against *L. donovani* elsewhere [42]–[44] and provide confidence that the assay can be used successfully to screen new compounds for activity against *Leishmania* amastigotes.

To this end, we progressed to testing a number of available NMT inhibitors, including those with advanced lead-like activity in *T. brucei* blood stream parasites, potent inhibitory activity against *L. major* NMT [31], [36] and the initial hits from the recent Pfizer HTS screen [35]. These were first tested against purified recombinant LdNMT to confirm their potency and then tested in the extracellular model described above, in which isolated splenic *L. donovani* amastigotes were incubated in the presence of inhibitor at 26°C. This method has the advantage of using host-derived amastigotes as the starting point as opposed to axenic amastigotes, which differ from intracellular amastigotes [17], [18], especially in their metabolism [18]. Conversely, the model could be problematic for general compound screening, introducing a bias towards compounds targeting parasite differentiation or promastigote replication. The final stage of testing used the new intracellular assay.

Focusing firstly on the TbNMT inhibitors DDD85646, DDD100887 and DDD86211 [31], [36], these compounds gave IC_50_ values of 4.4 nM, 20 nM and 7.3 nM respectively against *L. donovani* NMT. However, surprisingly, DDD85646 translated the least effectively from enzyme to cell, with a sharp drop in potency against *L. donovani* extracellular amastigotes, resulting in an EC_50_ of 8 µM.

Compounds DDD100887 and DDD86211 were more effective, generating EC_50_ values of 660 nM and 180 nM, respectively. Thus, the chemical modifications introduced into these compounds (moderation of their basicity or reduction in the number of hydrogen bond donors) resulted in an improved translation of enzyme to cell activity, perhaps due to better cellular penetration as compared to DDD85646 [36]. These results were partially reflected in the intracellular model data but the compounds were ∼10-fold (in the case of DDD100887) less active when compared to the purified NMT enzyme, the cellular target. This was the only compound for which a genuine dose-response curve could be plotted and an EC_50_ value calculated. Both DDD85646 and DDD86211 did not result in an expected dose-response relationship, precluding the EC_50_ calculation. Initially the compounds displayed cytotoxic effects before an “activity valley” was detected at higher concentrations. Both compounds were cytotoxic, at 45 µM, however, with all amastigotes cleared from the macrophages at this compound concentration. We currently have no rational explanation for the occurrence of this activity valley.

The remarkably good translation between NMT activity and cellular activity of DDD85646 observed in *T. brucei* blood stream parasites [31] was not observed for *L. donovani* in either the extra- or the intracellular model. Procyclic (insect) stages of *T. brucei* parasites were also much less sensitive to DDD85646 (pers. comm. H.P. Price), perhaps due to the different rates of endocytosis between the two parasite stages (with bloodstream trypanosomes having an unusually high rate [47]–[49]. Endocytosis rates are also slower in *Leishmania* species when compared to the bloodstream form of *T brucei*, although recent data suggest up-regulated endocytosis in *L. mexicana* amastigotes [50]. A similar lack of translation from enzyme to cellular activity has been reported for *L. major* CRK3 kinase inhibitors [51], [52], perhaps reflecting more general problems in targeting amastigote stages of *Leishmania*. These might include uptake and delivery of compounds into the macrophage, their chemical modification as a result of the acidic environment of the parasitophorous vacuole and/or their export from the phagolysosome via diffusion or active transport.

The second set of compounds tested in this study, derived from the Pfizer screen [35] were no better than the optimised Dundee derivative compounds in the extracellular amastigote assay. However, this analysis aided identification of issues to be addressed in a hit-to-lead development programme. Thus, IMP-0000195 (IC_50_ 76.5 nM, EC_50_ 12 µM) was the best enzyme inhibitor but also showed host cell toxicity and no selectivity. IMP-0000556 did not result in any leishmanicidal activity, probably due to insufficient potency - this is the compound with the least activity in the enzyme assay. More promisingly, IMP-0000083 and IMP-0000197 showed moderate cellular activity in the extracellular assay with no host cell cytotoxicity up to at least 45 µM. However, these two compounds showed no activity in the intracellular assay. Clearly, more work is required to improve both the potency and to optimise the physicochemical properties of these initial hits to improve cellular activity, developments that are currently in progress.

In summary, we report a robust assay for screening compounds against intracellular *Leishmania* amastigotes that should enable others to screen natural product or other libraries for identification of compounds active against the clinically-relevant life cycle stage. *Leishmania* NMT inhibitors are in early development but the compounds described here show good potential for further progression with respect to selectivity and cellular potency. In addition, methods that enhance compound delivery into both the parasitophorous vacuole and the intracellular parasites require further investigation. An optimised delivery system might increase the therapeutic “window”, thereby greatly improving the modest selectivity or potency observed so far.
